# Headache in essential thrombocythaemia

**DOI:** 10.1111/j.1742-1241.2012.02986.x

**Published:** 2012-08-14

**Authors:** R Frewin, A Dowson

**Affiliations:** 1 Pathology Department, Gloucester Royal HospitalGloucester, UK; 2 The King's Headache Service, King's College HospitalLondon, UK

## Abstract

Headache is frequently reported as one of the neurological manifestations of essential thrombocythaemia (ET) and other myeloproliferative neoplasms. It is associated with considerable morbidity; yet, it is a frequently under-recognised symptom. In patients with ET, headaches may be attributable to the disease, to the prescribed ET treatment, or unrelated to ET. The majority of headaches in ET are self-limiting and can be managed with standard headache therapies such as paracetamol, but it is vital that the clinician managing these conditions is able to recognise the headaches with a more sinister pathology. In this article, we will review the incidence and management of headaches in ET, whether they are primarily related to the disease or a result of its treatment. Identification of specific headache types in patients with ET may enable physicians to employ the most effective headache medication. This would enhance the patient-physician relationship, increasing patient compliance and thus reducing the risk of adverse outcomes.

**Review criteria**

A review of selected relevant papers focused on headache in ET. Relevant articles were identified by a literature search in PubMed. Further selection of articles was achieved by focusing on the following key points: incidence and prevalence of headache in ET; features of ET-related headaches; possible mechanisms for headaches in ET; treatment of ET-related headache; headache associated with treatment of ET.

**Message for the clinic**

Headaches are common in ET and may be related to the disease or its treatment. They are rarely sinister but clinicians should quickly assess headaches and reassure their patients by eliminating ‘red flag’ symptoms and signs. If headache is clearly temporarily related to the ET diagnosis, then low-dose aspirin therapy is frequently successful. If this fails, appropriate prescription of headache medication can lead to improved compliance with ET medication, resulting in preferable outcomes.

## Introduction

Headache is a common condition that affects almost the whole population at some time in their lives; it usually lasts for only a few hours and/or is easily remedied with over-the-counter medication such as paracetamol. However, headaches are believed to be disabling in approximately 18% of the UK population, with migraine (approximately 15%) (1) and chronic headache (approximately 3%) (2) accounting for notable loss of productivity and socio-economic burden. Headaches are believed to be under-diagnosed across Europe and the USA (3).

Essential thrombocythaemia (ET) is a clonal myeloproliferative neoplasm characterised by a sustained thrombocytosis of > 450 × 10^9^/l and the exclusion of reactive causes (4). Thrombohaemorrhagic complications, progression to myelofibrosis and transformation to acute myeloid leukaemia are the major causes of morbidity and mortality in patients with ET (5). Neurological symptoms including headache and numbness in the extremities are also frequently reported in patients with ET (6,7). Treatment is primarily focused on reducing the risk of thrombotic complications, and patients considered at high risk (i.e. > 60 years of age, or platelet count > 1500 × 10^9^/l, or with a history of thrombohaemorrhagic events) typically receive cytoreductive therapy such as hydroxycarbamide (HC; Hydrea®, Bristol-Myers Squibb, NY, USA), which has been proven to reduce thrombotic events in a randomised, controlled setting (8).

## Methods

Patients with ET frequently report headaches, but there remains a lack of detailed information describing how headaches should be managed in patients with ET. Here, we review the available literature on headache in ET. Relevant articles were identified by a literature search in PubMed. Further selection of articles was achieved by focusing on the following key points: incidence and prevalence of headache in ET; features of ET-related headaches; possible mechanisms for headaches in ET; treatment of ET-related headache; headache associated with treatment of ET. In addition, the authors’ clinical experience of headache in ET have been utilised to propose a schema to assist in the assessment and treatment of headache in patients with ET.

## Results

### Headache in ET

#### Incidence and prevalence

Episodes of neurological symptoms including headache, paresthesias and dizziness are frequently encountered as clinical manifestations in myeloproliferative neoplasms (6,7,9,10). There have been few studies investigating the prevalence of headache in patients with ET, but reports suggest between 4% and 39% of patients experience headache symptoms at some time during the course of the disease (6,10–13); this could mean that headaches are twice as frequent in patients with ET than in the general population. One of the few prospective studies noted that 5 of 37 patients newly presenting with ET complained of headache (14).

#### Features of ET-related headaches

As headache is a frequent symptom in the general population, it is important that the clinician can distinguish whether it is a primary problem, or a consequence of the ET or its treatment (secondary headache).

Unfortunately, headache in ET has not been well studied and remains poorly characterised. Much of the data recorded on headaches in patients with ET only meet standard adverse event reporting criteria (i.e. only the severity of the headache is documented). Classifications based on the International Headache Society (IHS) Classification of Headache Disorders (15), which recognises and outlines diagnostic criteria for several different types of headache, may provide greater insight into the nature of headaches in patients with ET (16). The diagnosis of chronic headache has evolved over time and remains a confusing issue. However, in practice, a headache is related to ET if the onset of this new symptom bears a close temporal relationship to the diagnosis of ET and resolves within 3 months of successful treatment (15). This may be due to either the use of low-dose aspirin therapy (7) or cytoreductive therapy (14). The available data suggest that ET-related headaches are usually intermittent (7). Pain is often described as dull, throbbing or pulsatile, with sudden onset and usually lasting for several hours (7,11,13). There have been reports of patients with ET experiencing headaches that feature transient visual disturbances, which bear a similarity to migraine aura (11,13). Jabaily et al. (11) reviewed 33 patients with ET: 13 patients reported headache and six reported visual disturbances (11). Michiels et al. (13) reported ‘transient cerebral or visual symptoms’ related to ET in 17 patients, 10 of whom reported headache. Differential diagnosis from migraine may be difficult, as ET-related headache symptoms can be very similar (7). However, it has been noted that symptoms of ET-related headaches occur suddenly, whereas in migraine symptoms tend to develop gradually over a period of time (7,11,13). There are no reports of neurological symptoms progressing to disabling stroke or permanent blindness; however, mild residual neurological sequelae may persist in some cases (7).

#### Possible mechanisms for headaches in patients with ET

In the prospective Jabaily study, neurological symptoms were found to coincide with periods of haematological relapse (11), which provides some evidence for a central role of platelets in the aetiology of headache in patients with ET. Possible causes of headache associated with platelet dysfunction include hypersensitivity of serotonin receptors (17) or increased plasma levels of serotonin (18,19). Headache has also been associated with increased levels of platelet adenosine diphosphate (18) and neuropeptide calcitonin gene-related peptide (20,21). The similarity in presentation of symptoms between ET-related headaches and migraine lends further credence to the suspected role of platelet dysfunction in headache in patients with ET and in those with migraine experience (18). Further evidence for the central role of platelet dysfunction comes from the study by Michiels (22), which reported that transient neurological symptoms (including headache) were often preceded or followed by erythromelalgia. Skin punch biopsies of patients affected by erythromelalgia demonstrated platelet-rich thrombi with the elevation of numerous markers of platelet activation, including raised β-thromboglobulin, platelet factor 4 and thrombomodulin. Platelet survival was also significantly reduced. Treatment with aspirin reduced these markers of platelet activation in line with normalisation of platelet survival in clinically responding individuals. Due to the similarity of response of both erythromelalgia and headaches to aspirin, a similar causative mechanism may be postulated.

Further studies support a microcirculatory disturbance in the aetiology of headaches in ET (6,10,28), which may be attributed to not only the thrombocytosis but also to the leukocytosis (23) or high red cell count (24). This was supported by the observation that treatment with low-dose aspirin is associated with relief from, and prevention of, headache symptoms (6,10,30). An association between leukocytosis and thrombotic risk has been made in a number of studies (25–27). To date, a direct link between leukocytosis and headache in ET has not been made.

Although headache has been identified as a symptom of anaemia in pregnant women (25), the role of haemoglobin levels in headache symptoms is unclear. A population-based cross-sectional study in Norway found a linear trend of decreasing prevalence of headache (p = 0.02) and migraine (p = 0.01) with decreasing haemoglobin levels (26). Headache prevalence was lower (odds ratio = 0.6, 95% confidence interval 0.4, 1.0) when haemoglobin was low (< 11.5 g/dl), compared with when haemoglobin was within normal ranges. However, a retrospective study of 70 patients with ET found no association between haemoglobin levels and the occurrence of neurological symptoms, including headache (6,10).

Another possible contributing factor to headache in patients with ET is increased levels of nitric oxide (NO), an important molecule in the regulation of cerebral and extra cerebral cranial blood flow. Headache and migraine have been associated with increased levels of NO (27–29), although NO response appears to be reduced in chronic myeloproliferative neoplasms (30).

#### Risks associated with headaches in ET

Patients with ET are at an increased risk of developing thrombohaemorrhagic complications. Thus, stratification of patients into risk groups defined by factors increasing their risk of complications, e.g. age > 60 years, previous ET-related thrombotic event or platelet count > 1500 × 10^9^/l, is recommended to define treatment strategies (4). Other retrospective studies have reported conflicting results on the role of hypertension, hyperlipidaemia, smoking and diabetes mellitus in contributing to the risk of major vascular complications (31,32), but international guidelines still recommend aggressive management of cardiovascular risk factors, particularly because ET-related headache can be attributed to elevated blood pressure in some cases (33). Recent evidence suggests that homozygosity for the JAK2 V617F mutation in ET is associated with a significantly increased incidence of cardiovascular events (34).

Patients may fear that a headache is indicative of, or a precursor to, a stroke; however, there is little evidence to suggest that this may be the case. Patients with ET should be reassured that headache is a common symptom of their condition that can be easily managed. In particular, it should be emphasised that headaches are rarely a precursor to a stroke or bleed. For example, in the PT1 study, the incidence of complications in patients with ET who were at high risk of vascular events and treated with low-dose aspirin plus either anagrelide or hydroxyurea was 5/809, intracerebral haemorrhage (ICH); 15/809, transient ischaemic attack and 16/809, cerebrovascular accident (35).

Clinicians managing patients with ET should still be aware of certain ‘Red Flag’ signs (36) or symptoms that indicate the possibility of an underlying sinister cause for their headaches. Worrying symptoms include an alteration in the headache pattern, with progression of both frequency and severity, headaches that awaken the patient from sleep or are of sudden and severe onset (the ‘thunderclap headache’) (3,15). Provocation of headache pain associated with exercise or coughing/sneezing is also significant. Any headaches developing after trauma should be investigated promptly, as should headaches with neurological signs, systemic upset and persistent or progressive vomiting (3,15). Billot (14) reported that 30% of new patients presenting with ET had neurological symptoms. However, the MRI scan was normal in those patients who had only subjective symptoms (including headache). Migraine in Primary Care Advisors (MIPCA) provides a diagnostic algorithm to identify potentially sinister headaches that require immediate follow-up (37).

### Treatment of ET-related headache

If the headache bears a clear temporal relationship to the diagnosis of ET and lacks any ‘Red Flag’ symptoms or signs, then low-dose aspirin alone should be tried in the first instance. There is a strong body of evidence that low-dose aspirin can effectively relieve the neurological symptoms in ET, such as headache and associated visual disturbances (7,13,14). If patients fail to respond to aspirin, then there is little evidence of benefit for combining the aspirin with clopidogrel or the use of Coumadin derivatives. In the event of aspirin failure, combined cytoreductive therapy and aspirin should be considered (14).

**Figure 1:**
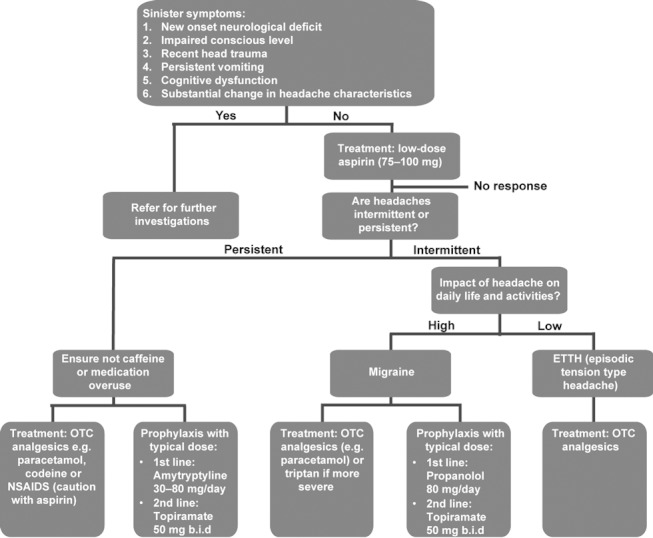
Management of ET-related headache

It is important to correctly identify the type of headache presenting in patients with ET, as different types of headache may require different approaches to management. We propose a schema to assist the general practitioner in the assessment and treatment of headache in patients with ET (see Figure 1). The schema presents common headache types that may be experienced by patients with ET.

Of primary importance is the exclusion of possibly sinister headaches (see Figure 1). Following this, when selecting an appropriate management strategy for headache in patients with ET, physicians should assess the frequency and duration of symptoms, along with the impact of headache on the patient’s daily activities. At this level in our schema, we classify headache as persistent or intermittent. In the general population, the majority of intermittent headaches have a low impact on the patient’s daily activities and are typically episodic tension type headache (ETTH). When intermittent headaches have a high impact on the patient’s daily activities, disrupting work, chores or social activities, they are more likely to be migraine. This distinction between ETTH and migraine is important, as the two conditions should be managed differently, for example, triptans may be used to treat migraine but are not a recommended treatment option for ETTH (see Figure 1). When headaches are persistent, it is critical to ensure this is not driven by caffeine- or medication-overuse. For example, patients using codeine regularly two or more times per week are at risk of developing medication-overuse headaches.

In the majority of patients with ET who report headache, symptoms are of mild-to-moderate severity (38). Standard mild analgesics such as paracetamol and/or low-dose aspirin should effectively manage headache symptoms in ET. Long-acting analgesics or daily prophylactic treatment may also warrant consideration in patients who experience headaches over several days or more, although care must be taken to avoid compounding symptoms with medication-overuse headache.

In cases of diagnostic difficulty or when the management of headache is problematic, patients should be referred appropriately.

### Patient-physician relationships

Patients with ET, particularly those who are diagnosed in their younger years, may be anxious about their condition and its symptoms. Physicians should take the time to listen to their patients, ensure that they feel that their concerns are being addressed, prescribe suitable headache medication and advise them to continue their regular ET medication. Patients who feel supported by their physicians have higher rates of adherence to their regular treatment (39), which in terms of ET is very important to prevent thrombohaemorrhagic complications.

### Headache associated with treatment of ET

While patients with ET may experience headaches as a result of their ET, some headaches these patients experience may be related to their ET treatment. In this section, we will discuss the main therapeutic options for ET and review the associated neurological adverse effects.

#### Hydroxycarbamide

HC is the first-line treatment for patients with ET. Starting doses are usually in the region of 15 mg/kg/day and dose adjustments are made to maintain a platelet count below 600 × 10^9^/l (below 450 × 10^9^/l in the UK) without lowering the white blood cell count below 4 × 10^9^/l. Headache is a common systemic side effect of HC treatment (40). HC has been shown to be involved in the production of NO in erythrocytes, via induction of cGMP (41). Furthermore, recent data have demonstrated that patients with ET receiving HC had significantly higher levels of NO derivatives compared with non-HC treated patients (42). It is believed that NO-production following HC treatment may contribute to the pharmacological properties of the drug (43). Given that patients with myeloproliferative neoplasms may have reduced plasma NO, increased NO levels resulting from HC treatment may result in vasodilation and may contribute to headache symptoms in these patients. Indeed, there is evidence from animal models that NO from HC affects cerebral vascular permeability (44).

#### Anagrelide

Anagrelide (Xagrid®, Shire Pharmaceuticals Ltd, Basingstoke, Hants, UK) is a potent platelet-reducing agent that selectively inhibits megakaryocyte differentiation in the bone marrow. Anagrelide is indicated in high-risk patients with ET who are intolerant to their current therapy or whose elevated platelet counts are not reduced to an acceptable level by their current therapy (45). Headache is the most common side effect associated with anagrelide, with up to 25–52% of patients with ET reporting at least one headache during clinical trials (38,46–50). As up to 39% of patients with ET experience headaches related to their disease, it is apparent that a further proportion of patients who receive anagrelide appear to experience ‘new’ headaches associated with anagrelide treatment. However, it is probable that there is a degree of crossover between these two populations as the rates of headache incidence are so variable between the ET (4–39%) (6,10–13) and anagrelide-treated (25–52%) (38,46–50) populations. Although *in vitro* data suggested that anagrelide is a potent inhibitor of phosphodiesterase III activity (51,52), these findings were only observed at doses substantially greater than normal treatment doses of anagrelide in humans (45). Nevertheless, physicians often believe that headaches in patients receiving anagrelide may be attributed to this vasodilator activity (40).

Results from clinical studies suggest that the incidence of headache associated with anagrelide reduces over time. In the Phase II, prospective Anagrelide Study Group trial, it was noted that patients complained of headache during the first 2 weeks of therapy and that the headaches typically lasted for 12 days (46).

In two studies conducted with anagrelide in patients with myeloproliferative neoplasms (predominantly ET), the incidence of headaches decreased with subsequent increases in treatment doses. In one study, the incidence of headaches was 34.2% during the first 3 months of treatment, which reduced to 5.7% thereafter (49). In another study, 25% of patients reported headache during the first month of treatment, which decreased to 11% during months 2–3 and 10% during months 4–6 (48). The incidence of headache is frequently dose related, so gradual dose titration is recommended to help to diminish these effects (45).

Good communication between physicians and patients is important during treatment with anagrelide. At the initiation of treatment, physicians should advise their patients that they may experience headaches associated with anagrelide. The physician should emphasise that the headaches will most probably only occur during the first 2 weeks of treatment and advise their patient to take paracetamol to treat the headache. While recognising that the headaches may be unpleasant for the patient, the physician should highlight the importance of continuing with anagrelide. In comparison with the risks associated with thrombosis or bleeding, a headache is a temporary, minor inconvenience and should be recognised as such. Following good practice, physicians should see their patients approximately 3 weeks after beginning anagrelide therapy, and can review any remaining headaches at this point. Headaches that have not abated by this time point should be assessed and treated comprehensively according to the schema in Figure 1.

#### Interferon-α (IFN-α)

Patients who progress on, or are unsuitable for, treatment with HC or anagrelide may be prescribed IFN-α. IFN-α is a biological response-modifying cytokine with both myelosuppressive and immunomodulatory effects. IFN-α, may be a particularly appropriate treatment for pregnant women (53) or those planning pregnancy as it is not associated with teratogenic effects or adverse effects on male fertility. Unfortunately, IFN-α therapy is associated with significant side effects that include flu-like symptoms (such as chills and fever) as well as fatigue, headache, diarrhoea and a range of other haematological and autoimmune symptoms (40,54). Approximately 30% of patients need to discontinue treatment with IFN-α as they cannot tolerate the adverse events (40). However, the development of pegylated forms of IFN-α has resulted in reports of improved short-term tolerability (55).

#### Further line therapy: busulphan, pipobroman, radioactive phosphorous (P32)

The incidence of headaches with the cytoreductive agents busulphan, pipobroman and P32 has not been well documented. These drugs are not currently used as first-line treatment for ET; however, their use concurrently or consecutively with HC has been associated with a significantly increased risk of transformation to an acute leukaemia (56). Thus, it is vitally important for the clinician to manage side effects such as headaches effectively with first-line agents to avoid having to switch to alternative treatments.

## Discussion

Headaches are common in the general population. Therefore, patients with ET will sometimes experience headaches that are unrelated to either their haematological condition or its treatment. Headaches are widely misunderstood, misdiagnosed and mistreated. Furthermore, they have not been sufficiently recognised as an issue for patients with ET. Headaches should be assessed in their own right and the clinician should not necessarily attribute a headache to ET or its treatment. Although most headaches in patients with ET are benign, physicians should familiarise themselves with those headaches that may be a cause for concern and what to do in these situations. Most importantly, physicians should appreciate and understand the impact that headaches can have on their patients’ morbidity and compliance.

In the general population, general practitioners routinely see and treat patients with headaches. As many headaches reported by patients with ET will be similar in nature to those reported by the general population, haematologists treating patients with ET should follow the same structured approach to diagnosis and treatment that primary care physicians use for their patients. However, if the haematologist is in any doubt (e.g. in cases of diagnostic difficulty, when a patient presents with a type of headache that is less common or when the management of headache is problematic), patients should be referred to an appropriate specialist.

It is recognised that not all patients with ET achieve optimal platelet levels with current treatments. However, it should be considered that poor responses are sometimes directly related to poor compliance. As with any other chronic condition, compliance rates with ET are known to deteriorate over time. Furthermore, patients with ET may stop taking their medication if they believe that their headaches are caused by it, whether they are correct or not. Non-compliance with ET medication due to the regular, mild irritation of a headache can have serious consequences for patients. Patient-physician relationships are very important in compliance issues, and patients who feel listened to and have physicians who address their concerns tend to report better quality of life than those who do not. Patients who perceive their quality of life in a positive light may also be more compliant with their medication.

## Conclusion

Although patients with ET frequently report headaches that they fear may be a precursor to stroke, there is little evidence to support this perception. In fact, such headaches are rarely sinister and, if guidelines are followed, diagnosis should be straightforward. Physicians should try to correctly identify the type of headache based on clinical characteristics, so that the most appropriate management strategy can be employed. In the case of headache associated with anagrelide treatment, the incidence of this can be minimised by gradual dose titration at onset of treatment. It is important for physicians to reassure patients that headache in ET can usually be easily managed with standard headache treatments such as paracetamol. Physicians should emphasise that patients should not stop taking their regular medication as premature discontinuation of ET treatment may put patients at increased risk of thrombotic events. Indeed, if headaches are well managed, the likelihood of patients showing poor compliance with early-line treatments is reduced, which will enable patients to stay on treatments that have fewer long-term risks. Given the lack of detailed information on headaches experienced in patients with ET, further research may be appropriate, which could be carried out using patient diaries to understand the nature, frequency of incidence and severity of the headaches to enable their characterisation and appropriate treatment.
